# The Heterotrimeric Laminin Coiled-Coil Domain Exerts Anti-Adhesive Effects and Induces a Pro-Invasive Phenotype

**DOI:** 10.1371/journal.pone.0039097

**Published:** 2012-06-19

**Authors:** Patricia Santos-Valle, Irene Guijarro-Muñoz, Ángel M. Cuesta, Vanesa Alonso-Camino, Maider Villate, Ana Álvarez-Cienfuegos, Francisco J. Blanco, Laura Sanz, Luis Álvarez-Vallina

**Affiliations:** 1 Molecular Immunology Unit, Hospital Universitario Puerta de Hierro, Majadahonda, Madrid, Spain; 2 Structural Biology Unit, CIC bioGUNE, Parque Tecnológico de Bizkaia, Derio, Spain; 3 IKERBASQUE, Basque Foundation for Science, Bilbao, Spain; University of Birmingham, United Kingdom

## Abstract

Laminins are large heterotrimeric cross-shaped extracellular matrix glycoproteins with terminal globular domains and a coiled-coil region through which the three chains are assembled and covalently linked. Laminins are key components of basement membranes, and they serve as attachment sites for cell adhesion, migration and proliferation. In this work, we produced a recombinant fragment comprising the entire laminin coiled-coil of the α1-, β1-, and γ1-chains that assemble into a stable heterotrimeric coiled-coil structure independently of the rest of the molecule. This domain was biologically active and not only failed to serve as a substrate for cell attachment, spreading and focal adhesion formation but also inhibited cell adhesion to laminin when added to cells in a soluble form at the time of seeding. Furthermore, gene array expression profiling in cells cultured in the presence of the laminin coiled-coil domain revealed up-regulation of genes involved in cell motility and invasion. These findings were confirmed by real-time quantitative PCR and zymography assays. In conclusion, this study shows for the first time that the laminin coiled-coil domain displays anti-adhesive functions and has potential implications for cell migration during matrix remodeling.

## Introduction

Laminins are a family of extracellular matrix (ECM) glycoproteins localized in the basement membrane that, in addition to having structural roles have been shown to regulate many cellular processes, such as cell migration, differentiation, and proliferation. Laminins are heterotrimeric molecules consisting of variable associations of each one α, β, and γchains assembled in a coiled-coil structure [1]. The α1, α2, α3B, and α5 laminins possess three short arms with globular LN domains at the N termini of each of the three subunits. In contrast, α4 laminins possess only two short arms because of truncation of the α-subunit short arm. All α-chain laminins contain 5 globular LG domains at their C-termini [2].

Laminins have multiple binding partners. However, most of the binding sites map to regions distinct from the coiled-coil domain. The current interpretation is that cell-binding sites are located on adjacent LN or LG globular domains, which, for correct folding, require the presence of the adjacent helical rod [3]. To date, structure-function relationships have come largely from studies with proteolytic fragments, recombinant globular domains and synthetic overlapping peptides covering the entire sequence of individual laminin subunits [4]. However, it is evident that with these approaches identification of new functional regions in highly structured areas is not possible.

Using an indirect phage display-assisted mapping strategy to preserve protein structure, we have reported previously that the coiled-coil domain of laminin-1 (LM111, α1β1γ1-subunit composition) contains a cell-binding site [5]. According to our data, the adhesion motif is formed by residues contributed by both α1 and γ1-chains, and is located in the middle part of the coiled-coil domain. To our knowledge, besides this interaction and the binding of agrin [6] no other interactions between the laminin coiled-coil domain and other proteins have been described.

Therefore, a detailed characterization should be of particular importance to assign precise functions to laminin coiled-coil domain, beyond oligomerization. To facilitate these studies, we have produced a recombinant fragment corresponding to the laminin coiled-coil by transfecting 293 cells with cDNAs coding for truncated α1-, β1-, and γ1-chains and studied its properties with respect to cell adhesion, motility and gene expression.

## Materials and Methods

### Antibodies and Reactives

The monoclonal antibodies (mAbs) used included 9E10 (Abcam, Cambridge, UK) specific for c-myc tag, BMG-His-1 (Roche Applied Science, Germany) specific for His tag, 12CA5 (Abcam) specific for HA tag, M2 (Sigma-Aldrich, St Louis, MO, USA) specific for FLAG tag, and biotin-conjugated 4D11 (Millipore Corporation, Billerica, MA, USA) specific for His tag. The polyclonal antibodies used included a rabbit anti-laminin (Sigma-Aldrich), a rabbit anti-fd-bacteriophage (Sigma-Aldrich), an HRP-conjugated donkey anti-rabbit IgG (GE Healthcare, Uppsala, Sweden), an HRP-conjugated goat anti-mouse IgG (Sigma-Aldrich), an IRDye800-conjugated donkey anti-mouse IgG (Rockland Immunochemicals Inc., Gilbertsville, PA, USA), a PE-conjugated goat anti-mouse IgG (Jackson ImmunoResearch Europe, Suffolk, UK), FITC-conjugated goat anti-mouse IgG (Sigma-Aldrich), and a FITC-conjugated goat anti-rabbit IgG (Jackson ImmunoResearch Europe). Laminin extracted from the Engelbreth-Holm-Swarm (EHS) mouse tumor was from Becton Dickinson Labware (Bedford, MA, USA), human plasma fibronectin, HRP-conjugated Protein A, and HRP-conjugated streptavidin were from Sigma-Aldrich.

### Cells and Culture Conditions

HT1080 cells (human fibrosarcoma; CCL-121), and HEK-293 cells (human embryo kidney epitheli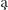 CRL-1573) were obtained form the American Type Culture Collection (Rockville, MD, USA) and were cultured in DMEM supplemented with heat-inactivated 10% (vol/vol) fetal calf serum (FCS), 2 mM L-glutamine, and penicillin/streptomycin (all from Invitrogen Life Technologies, Carlsbad, CA, USA). FreeStyle 293-F cells were cultured in FreeStyle™ 293 expression medium (Invitrogen Life Technologies) in humidified 8% CO_2_ atmosphere at 37°C.

### Construction of Vectors

The mouse truncated laminin α1-chain (from amino acid 1409 to 2145, accession number ENSMUSP00000043957 in the Ensembl data base) was amplified in 15 cycles by PCR using the Ecotaq-plus DNA polymerase High Fidelity, according to the manufacturer’s instruction (Ecogen, Barcelona, Spain), with the primers 1 and 2 (Table S1) using 0.1 ng of plasmid pCIS mouse laminin α1 (a kind gift from Dr. Peter Yurchenco, Robert Wood Johnson Medical School, Department of Pathology, Piscataway, NJ, USA), and ligated into the pCR2.1-TOPO vector using the TOPO TA Cloning Kit (Invitrogen Life Technologies) and called pCR2.1-tα1. The sequence was verified using primers 3, 4, 5 and 6 (Table S1). A 2207 bp HindIII/NotI fragment derived from the plasmid pCR2.1-tα1 was introduced into the HindIII/NotI site of pSECTAG2B (Invitrogen Life Technologies) resulting in pSECTAG2B.tα1 vector. An oligo pair (primers 7 and 8) containing the FLAG-tag sequence (DYKDDDDK) was ligated into the NotI/XbaI site of pcDNA3.1/Hygro (+) (Invitrogen Life Technologies), resulting in pcDNA3.1/Hygro.FLAG. A 3208 bp BglII/NotI fragment derived from the plasmid pSECTAG2B.tα1was cloned in pcDNA3.1/Hygro-FLAG to obtain the plasmid pcDNA3.1/Hygro.tα1.FLAG.

The mouse truncated laminin β1-chain (from amino acid 1075 to 1834), accession number ENSMUSP00000002979 in the Ensembl data base), was amplified in 15 cycles by PCR using the Ecotaq-plus DNA polymerase High Fidelity, with the primers 9 and 10 (Table S1) using 0.1 ng of plasmid pCIS mouse laminin β1 (Dr. Peter Yurchenco), and ligated into the pCR2.1-TOPO resulting in pCR2.1-tβ1 The sequence was verified using primers 11, 12 and 13. A 2291 bp BamHI/NotI fragment derived from the plasmid pCR2.1-tβ1 was introduced into the BamHI/NotI site of pSECTAG2B resulting in pSECTAG2B.tβ1.

The mouse truncated laminin γ1-chain (from amino acid 928 to 1609), accession number ENSMUSP00000027752 in the Ensembl data base) was synthesized by GENEART AG (BioPark Regensburg, Germany). A 2007 bp DNA fragment containing the HA-Tag sequence (YPYDVPDYA) was introduced into HindIII/NotI site of vector pSECTAG2B to obtain the plasmid pSECTAG2B.tγ1. A 2605 bp NdeI/NotI fragment derived from the plasmid pSECTAG2B.tγ1 was introduced into the NdeI/NotI site of pcDNA3.1/Neo (+) (Invitrogen Life Technologies) to obtain the plasmid pcDNA3.1/Neo.tγ1.

**Figure 1:**
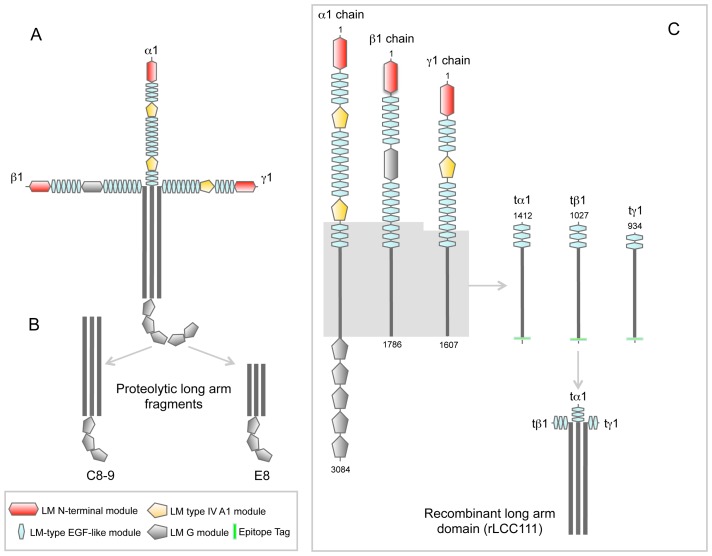
Schematic representation of structure of laminin-111 (A), and proteolytic fragments derived from the long arm (B) by elastase treatment (E8) and by cathepsin G treatment (C8-9). Schematic representation of the domain organization of laminin-111 and the recombinant laminin coiled-coil domain (rLCC111) used in this work (C). Amino acid positions are indicated according to the numbering of [44]–[46].

### Cell Transfections and Purification of Recombinant Proteins

HEK-293 cells were transfected with plasmids pSECTAG2B.tα1, pSECTAG2B.tβ1 or pSECTAG2B.tγ1 using Superfect (QIAGEN GmbH, Hilden, Germany). Supernatants were collected at 48 h and analyzed by ELISA and western blotting using anti-c-myc mAb. For simultaneous expression 293-F cells were transfected with plasmids pcDNA3.1/Hygro.tα1.FLAG, pSECTAG2B.tβ1 and pcDNA3.1/Neo.tγ1 using 293 fectin (Invitrogen Life Technologies) and cultured in Free-Style serum-free medium. Supernatants were collected at 48 h and analyzed by ELISA and western blotting using anti-FLAG, anti-c-myc mAb, or anti-HA mAbs. For purification by immobilized metal affinity chromatography (IMAC), the collected media was loaded onto a HisTrap HP 1 ml column (GE Healthcare), previously equilibrated in 0.5 M NaCl, 5 mM imidazole (pH 7.4) and eluted with 0.5 M NaCl, 200 mM imidazole (pH 7.4) using an ÄKTA Prime plus System (GE Healthcare). The purified rLCC111 dialyzed against PBS, quantified by Coomassie Plus™ Protein assay (Thermo Fisher Scientific Pierce Rockford, IL) and analyzed by SDS-PAGE under reducing conditions using 4–12% gradient polyacrylamide gels (Invitrogen Life Technologies). For western blotting, protein samples were blotted onto nitrocellulose membranes (Invitrogen Life Technologies) and reacted with specific anti-tag mAb, followed by incubation with an IRDye800-conjugated donkey anti-mouse IgG. Visualization and quantitative analysis of protein bands were carried out with the Odyssey® infrared imaging system (LI-COR Biosciences, Lincoln, NE, USA).

### Multi-angle Laser Light Scattering (MALLS)

Static light scattering experiments were performed at room temperature using a 7.8 mm×300 mm WTC-100S5 Size Exclusion Chromatography column (Wyatt, Tech. Corp., with a 50,000–7,500,000 Da separation range) attached in-line to a DAWN-HELEOS light scattering detector (Wyatt). The column was equilibrated with running buffer (PBS+0.03% NaN_3_, 0.1 µm filtered), and the system was calibrated using bovine serum albumin (BSA; Sigma-Aldrich). Samples of 120 µL of rLCC111 (at 0.1 mg/mL) were injected into the column at a flow rate of 0.5 mL/min. Data acquisition and analysis were carried out using ASTRA software (v 5.3.4.19, Wyatt).

**Figure 2:**
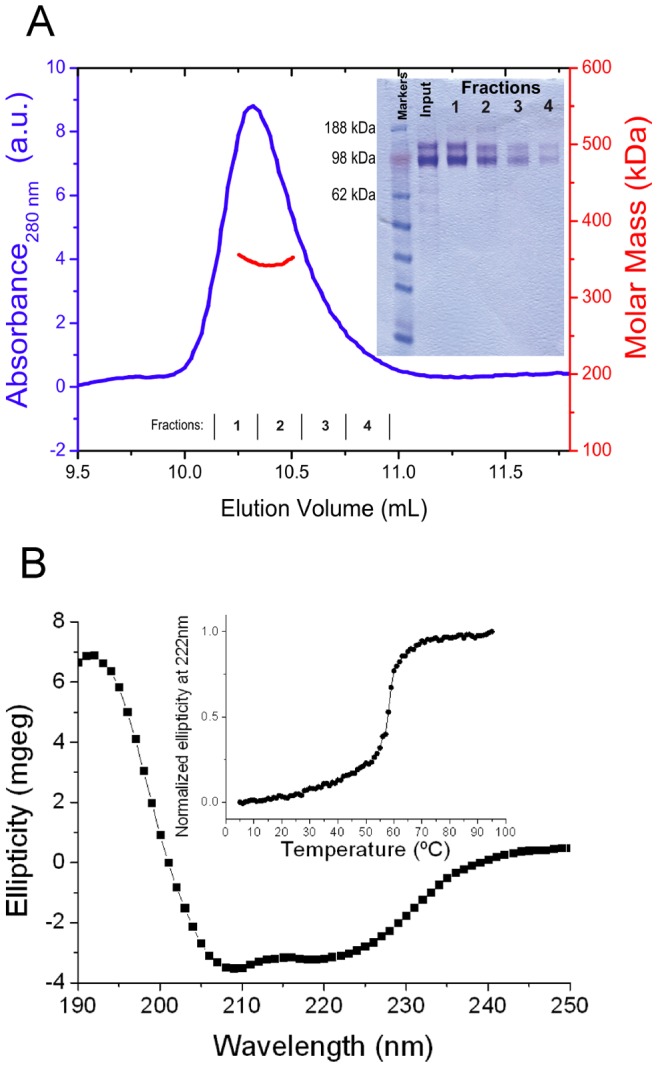
Structural analysis of rLCC111. Gel filtration chromatogram profile, as measured by UV absorbance at 280 nm (blue trace), and molar mass (red trace), as measured by MALLS (only the data at the central part of the peak is shown, and can be read at the right hand axis, in red color) The mass measured at the centre of the peak is 335 kDa (A). The inset shows a coomassie stained SDS-PAGE under reduction conditions of the input and the indicated separately collected fractions. Circular dichroism spectrum recorded at 25°C and thermal denaturation measured by the change in the ellipticity at 222 nm (B).

### Circular Dichroism (CD)

Circular dichroism measurements were performed with a Jasco J-810 spectropolarimeter. The spectrum was recorded on a protein sample at 0.18 mg/mL in PBS using a 0.01 cm path length quartz cuvettes at 25°C. Thermal denaturation was recorded on a protein sample at 0.03 mg/mL in PBS using a 0.2 cm path length quartz cuvette. Protein unfolding was induced by increasing temperature at a rate of 1°C/min (using a programmable Peltier thermoelectric) and measuring the ellipticity at 222 nm.

### Specific ELISA

Maxisorp 96-well plates (Nunc, Roskilde, Denmark) were coated overnight at 4°C with 1 µg/well of anti-c-myc, anti-FLAG or anti-HA mAb. After washing, plates were blocked with 2% PBS-BSA for 1 h at 37°C. Blocking solution were removed and supernatant from single transfected HEK-293 cells or triple transfected 293-F cells was added for 1 hour at room temperature. After washing 100 µl of rabbit anti-laminin antibody was added for 1 hour at RT. After washing HRP-conjugated donkey anti-rabbit IgG was added and incubated for 1 additional hour at RT. After another washing the chromogenic substrate 3, 3′, 5, 5′-tetramethyl benzidine (TMB; Sigma-Aldrich) was added and the absorbance was measured at 450 nm. For direct ELISA plates were coated overnight at 4°C with laminin-111 (1 µg/well) or rLCC111 (1 µg/well). After washing and blocking with 2% PBS-BSA, 100 µl of rabbit antibody (anti-laminin or anti-fd-bacteriophage) or purified scFv antibody (L36 [7] or 2.15 [8]) (0.5 µg/well) were added for 1 hour at room temperature. After washing HRP-conjugated donkey anti-rabbit IgG or HRP-conjugated Protein A was added for 1 hour at RT. For sandwich ELISA plates were coated with 1 µg/well of anti-c-myc, anti-FLAG or anti-HA mAb. After washing and blocking LM111 or rLCC111 was added for 1 hour at RT. After washing biotin-conjugated anti-His mAb was added for 1 hour at RT followed by HRP-conjugated streptavidin.

**Figure 3:**
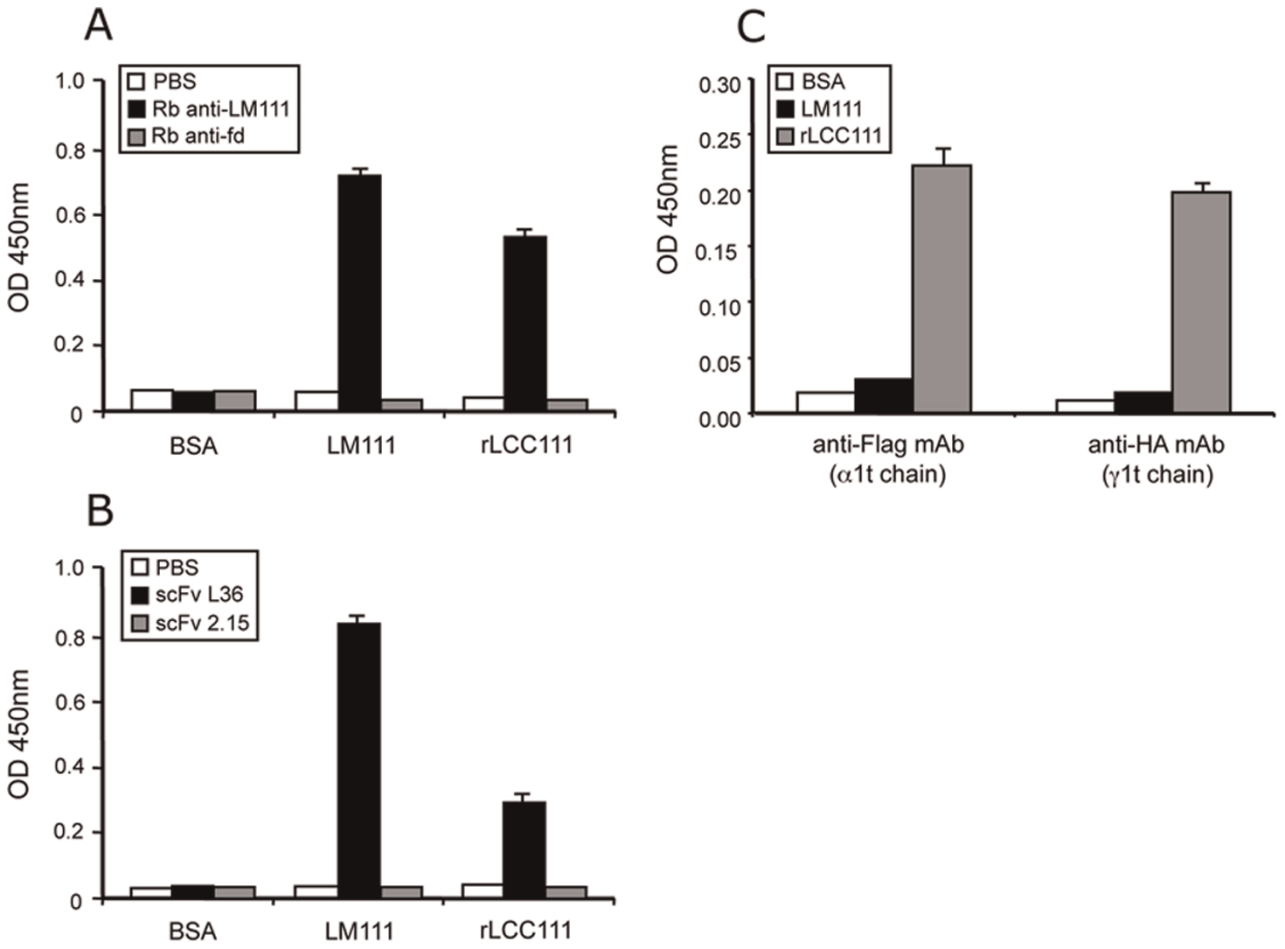
ELISA to assay binding of rabbit anti-laminin antibody (A) and the anti-laminin scFv antibody L36 (B) to plastic coated with LM111 or with rLCC111. As control, we used rabbit anti-fd-bacteriophage antibody (A) and the anti-p32 scFv antibody 2.15 (B). ELISA to demonstrate the presence in the same rLCC111 molecule of two truncated laminin chains, tα1 and tβ1 or tγ1 and tb1 (C).

### Immunofluorescence

The binding of the LM111 or rLCC111 to cell surface receptors in HT1080 cells was studied by immunofluorescence. HT1080 cells in suspension were incubated (30 minutes at 4°C) with LM111 or rLCC1111 (27 µg/ml). After washing cells were treated with a rabbit anti-laminin antibody or an anti-His mAb, and appropriate dilutions of FITC-conjugated goat anti-rabbit IgG or PE-conjugated goat anti-mouse IgG. Samples were analyzed with EPICS XL flow cytometer (Coulter Electronics, Hialeah, FL, USA). Alternatively, HT1080 cells were plated onto a Lab-Tek chamber slide (Nunc) in complete medium and allowed to attach and spread overnight. Then they were washed once with serum-free medium, and incubated (60 minutes at 4°C) with LM111 or rLCC111 (50 µg/ml) in PBS 1% BSA. After washing with PBS cells were fixed with 3% paraformaldehyde in PBS (15 minutes, room temperature) and washed with PBS. Primary antibodies (rabbit anti-laminin antibody or anti-His mAb) were added in PBS 1% BSA and incubated overnight at 4°C. After one wash with PBS and two with PBS 1% BSA, cells were stained by adding FITC-conjugated goat anti-rabbit IgG or FITC-conjugated goat anti-mouse IgG for 30 minutes at 4°C. After extensive rinsing with PBS, nuclei were counterstained with TO-PRO-3 (Invitrogen Life Technologies). Images were acquired using a Leica confocal microscope TCS-SP5 (Leica Microsystems, Mannheim, Germany).

### Cell Adhesion Assay

96-well microtiter plates (Corning Costar; Cambridge, MA, USA) were coated overnight at 4°C with LM111 (1 µg/well), rLCC111 (1 µg/well) or PBS. After washing plates were blocked with 3% BSA-DMEM for 1 hour at 37°C. Monodispersed suspensions of HT1080 cells were obtained using enzyme-free cell dissociation buffer (Sigma-Aldrich). Aliquots of 10^4^ cells were loaded per well in serum-free medium and incubated for 30 minutes in humidified 5% CO_2_ atmosphere at 37°C. After washing 100 µl of substrate CellTiter-Glo (Promega, Madison, WI) were added per well, and the bioluminescence measured using a Tecan Infinite F200 plate-reading luminometer (Tecan Trading AG, Switzerland).

### Cell Rounding Assay

96-well microtiter plates (Corning Costar) were coated overnight at 4°C with laminin-111 (1 µg/well), purified rLCC111 (1 µg/well), or PBS. After washing, wells were blocked with complete medium for 1 hour at 37°C. Monodispersed suspensions of HT1080 cells were obtained using enzyme-free cell dissociation buffer, seeded at 5×10^3^ cells/well and incubated for different periods of time in humidified 5% CO_2_ atmosphere at 37°C. For quantification of cell displaying rounded morphology three random fields were observed with phase-contrast microscopy (magnification×200) and cells were scored as rounded or spread.

### F-actin Staining

HT1080 cells were plated onto Lab-Tek chamber slides coated overnight at 4°C with laminin-111 (1 µg/well), purified rLCC111 (1 µg/well), or PBS. After a 3 hour incubation period in humidified 5% CO_2_ atmosphere at 37°C, cells were fixed with 2% paraformaldehyde, permeabilized with 0.1% Triton X-100, and washed twice with 1% BSA in PBS. The cells were incubated with Alexa Fluor 594 Phalloidin (0.5 µmol/L, Invitrogen Life Technologies) for 20 minutes to detect F-actin, followed by rinsing with 1% BSA in PBS. Images were captured using a Leica confocal microscope TCS-SP5 (Leica Microsystems).

**Figure 4:**
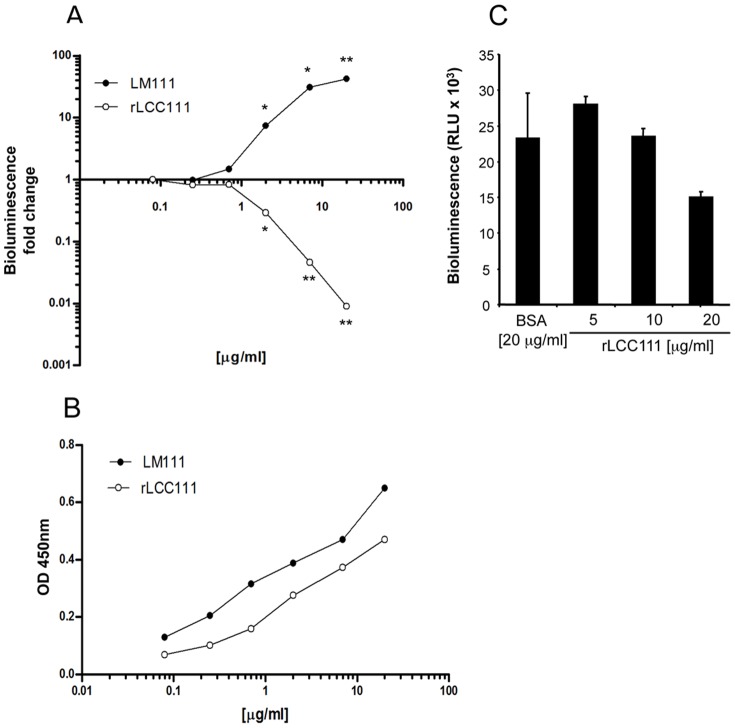
rLCC111 substrates do not support cell attachment. Adhesion of HT0180 cells to plastic-coated LM111 or rLCC111 (ranging from 0.08 to 20 μg/ml), or BSA was measured by bioluminescence. (A). Data are plotted as the log of fold change in adhesion relative to BSA. The coating efficiency of LM111 and rLCC111 was monitored by ELISA using a polyclonal anti-laminin antibody (B). Soluble rLCC111 (5 to 20 mg/ml) inhibits adhesion of HT1080 cells (C) to plastic immobilized intact LM111 (10 mg/ml). Data shown are from a representative experiment out of three independent ones. (*, p<0,05; **, p<0,005).

### Microarray Analysis

HT0180 cells were grown for 24 hours after seeding on 24-well plates coated with BSA, or rLCC111 (1 µg/well), and then total RNA was extracted using a Qiagen RNeasy Micro kit. RNA was quantified by NanoDrop spectrophotometer (Thermo Scientific) and checked for integrity on a Bioanalyzer 2100B (Agilent Technologies, Santa Clara, CA, USA). Double stranded cDNA was synthesized from 250 ng of total RNA using Ambion® WT Expression kit (Applied Biosystems, Carlsbad, CA, USA). After cDNA purification, this DNA was used as template for the *in vitro* transcription. The obtained cRNA was fragmented and hybridized to the GeneChip® Human Gene 1.0ST array (Affymetrix, Santa Clara, CA, USA) for 16 h at 45°C. Hybridized microarrays were washed and stained with a streptavidin-phycoerythrin conjugate in a “GeneChip® Fluidics Station 450”. All these procedures were carried out as suggested by the manufacturer at the Universidad Complutense de Madrid Genomics and Proteomics Core Facility. Hybridized cRNA was finally identified by the fluorescence signal in a “GeneChip® 3000” scanner. The CEL files generated from the scanning were converted to gene expression signals using RMA Algorithm [9] in Affymetrix® Expression Console™. Subsequent analyses were performed with Babelomics gene expression and functional analysis Suite version 4.2 (www.babelomics.org). Limma [10] was used for differential expression analysis. P-values were corrected by calculating the False Discovery Rate (FDR). The microarray data produced in this analysis are deposited in NCBÍs Gene Expression Ómnibus [11] and are accessible through GEO Series accession number GSE32896 (http://www.ncbi.nlm.nih.gov/geo/query/acc.cgi?acc=GSE32896).

### Functional Association Analysis

The list of differentially expressed genes was uploaded into the Database for Annotation, Visualization and Integrated Discovery v6.7 (DAVID; http://david.abcc.ncifcrf.gov/) [12], [13] to determine differentially regulated pathways using the full human genome as reference background. Data were analyzed in the “Functional Annotation Clustering” tool for Panther Molecular Function (MF) and Biological Process (BP) Gene Ontology (GO) terms.

### Real-time Quantitative PCR (qPCR)

cDNA from HUVEC or HT1080 cells was derived 250 ng of total RNA by random primed reverse transcription using SuperScript® VILO cDNA Synthesis Kit (Invitrogen Life Technologies) according to the recommended protocol. Primers for BLID, MMP2, MMP13, IL-24, SSP1, VCAN, WNT5A, GPNMB, MGP, OCR1, and SDHA genes were designed using the LightCycler Probe Design Software 2.0 (Roche Applied Science) and synthesized by Roche Applied Science. Primer pairs were designed for each of these genes to amplify products of 86–95 bp. Real-time PCR was done in a LightCycler 480 apparatus (Roche Applied Science) using the LightCycler 480 SYBR Green I Master kit (Roche Applied Science) under the following cycling conditions: initial denaturation of 3 min during which the well factor was measured, 45 cycles of 10 s at 95°C followed by 7s at 59°C. Fluorescence was measured during the annealing step in each cycle. After amplification, melting curves with 80 steps of 15 s and 0.5°C increase were performed to monitor amplicon identity. The relative expression of each mRNA was calculated by DC_T_ method (where DC_T_ is the value obtained by subtracting the C_T_ value of the internal loading control gene SDHA mRNA from the C_T_ value of the target mRNA). The amount of the target relative to the SDHA mRNA was expressed as 2^–(ΔCT)^. Primers are shown in Table S2.

### Gelatin Zymography

Conditioned media from HT1080 cells cultured in 96-well plates coated with BSA, laminin 111 or rLCC111 in serum-free media were analyzed for gelatine degrading activity by electrophoresis in 10% Pre-Cast polyacrylamide gels copolymerized with 1 mg/mL gelatin according to the manufacturer’s instructions (Invitrogen Life Technologies). Gels were stained with Coomassie brilliant blue and destained. The amounts of proenzyme and active metalloproteinase were analyzed by densitometry scanning of the gel.

**Figure 5:**
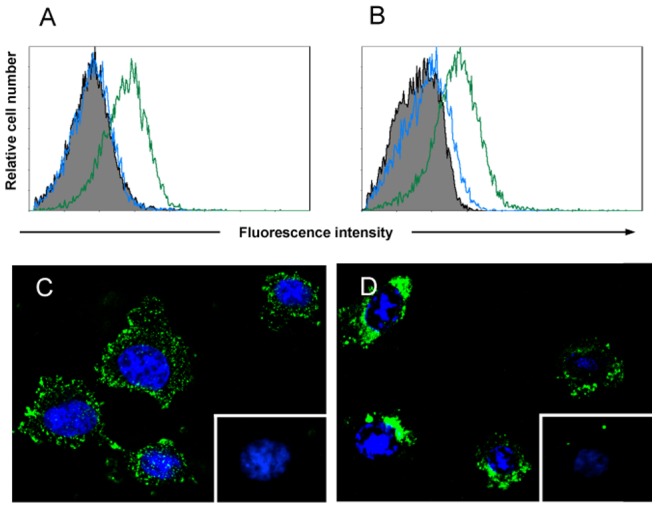
FACS profiles and representative microscopy images of HT1080 cells labeled with LM111 (A, C) or rLC111 (B, D), followed by incubation with rabbit anti-laminin antibody for the detection of LM111 (green line in FACS histogram A, and C) or anti-His mAb for the detection of rLC111 (green line in FACS histogram B, and D). To rule out the binding of anti-laminin antibody to endogenous laminin, HT1080 cells were incubated with the anti-laminin antibody in the absence of added LM111 (blue line in FACS histogram A, and inset C). A similar control was performed with the anti-His mAb in the absence of added rLC111 (blue line in FACS histogram B, and inset D).

## Results

### Design and Expression of Truncated Laminin α1-, β1-, and γ1-chains

Most proteolytic or recombinant laminin fragments generated to date correspond to N-or C-terminal portions of the molecule, and only two of these fragments (E8, C8-9) contain partial coiled-coil segments [14], [15], in both cases associated with three globular LG modules (Fig. 1). To precisely study the biological role of the laminin coiled-coil (LCC) we constructed recombinant cDNA fragments encoding the entire LCC of the α1-, β1-, and γ1*-*chains. Since the natural short variants of the laminin α-chain (α3A and α4) contain some laminin EGF-like (LE) modules in the N-terminal part, we decided to include three LE modules in the truncated α1 and β1 constructs, and two in the truncated γ1 construct (Fig. 1).

To study the fate of individual truncated laminin chains in the absence of their normal partners, we selected a transfectable cell line with low endogenous laminin chain expression. It has been previously shown that conditioned medium from wild-type HEK-293 cells did not react in western blots with a polyclonal anti-laminin antibody [16], [17]. The transfected truncated laminin α1 chain (tα1) could be secreted when expressed alone, but secretion of the myc-tagged truncated laminin β1- and γ1-chains (tβ1 and tγ1) chains was lower (Fig. S1A). Similar results were obtained by ELISA; in which anti-myc mAb was coated onto plates and bound secreted c-myc-tagged recombinant truncated laminin chains were detected with a polyclonal anti-laminin antibody (Fig. S1B).

By contrast 293-F cells transfected simultaneously with a Flag-tagged tα1 chain expression plasmid, a c-myc-tagged tβ1 chain expression plasmid, and a HA-tagged tγ1 chain expression plasmid secreted higher amounts of all three chains to the medium (Fig. S1C). Under reducing conditions, a broad band at around 110 kDa was detected with anti-myc mAb, whereas two major bands were detected with anti-Flag (135 and 125 kDa), and anti-HA (110 and 80 kDa) mAbs. The predicted molecular masses for tα1, tβ1, and tγ1 chains are 84, 88, and 81 kDa, respectively. Laminins are heavily glycosylated, which may account for the higher MW observed in SDS-PAGE. ELISA further confirmed the presence of secreted truncated laminin chains with anti-Flag, anti-c-myc or anti-HA mAbs (Fig. S1D).

**Figure 6:**
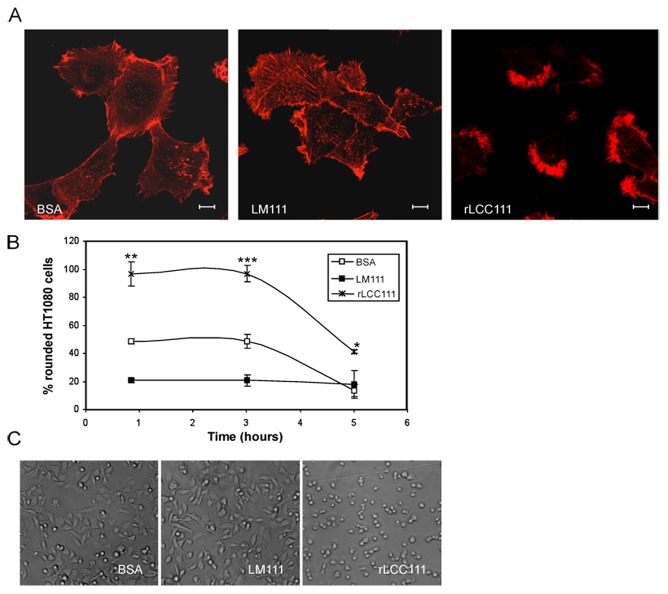
Morphology of HT1080 cells cultured on tissue culture plates coated with BSA, intact LM111 or rLCC111 (10 mg/ml). Cells were allowed to attach for 6 hours, followed by fixation and staining with Alexa Fluor 594 phalloidin (A). Scale bar is 10 µm. Spread and non-spread cells were counted and percentages of non-spread cells are indicated (B). Spread cells were defined as large cells with extensive visible lamellipodia, whereas non-spread cells were defined as round cells with little or no membrane protrusions. Spread and non-spread cells were counted in four high-power fields and represented as mean±SD for each condition and time point. The data were evaluated by *t* test and were considered to be statistically significant when *p*≤0.05. (*, *p*<0.05; **, *p*<0.005, ***, *p*<0.005). Representative phase-contrast images are shown from one of three independent experiments (C).

### Purification and Characterization of the Recombinant Laminin Coiled-coil Domain

Recombinant LCC domain (rLCC111) was purified from conditioned medium of triple-transfected 293-F cells by IMAC, and eluted as a single homogeneous peak. On SDS-PAGE under reducing conditions, the protein migrated as two major bands of 139 and 107 kDa, which reacted on western blots with anti-FLAG, anti c-myc and anti-HA mAbs (Fig. S2). This result indicates that the truncated chains assemble into a multimeric structure.

The trimeric nature of the assembly was confirmed by MALLS measurements. As seen in Figure 2A, the protein eluted from the column as a single symmetric peak with a mass of 335 kDa calculated from the light scattering measurements. However, the elution volume is smaller than expected for a molecule of this size. If this elution volume is used to calculate the molar mass based on the calibration of the column with a set of molecular weight markers, then an apparent mass of 700 kDa is obtained (data not shown). The reason for this discrepancy is the different effect of the molecular shape on the light scattering and on the elution volume. While elongated or disordered proteins elute from size exclusion columns at smaller volumes than globular proteins of the same mass (thus yielding apparent masses larger than real ones) the light scattering data analysis is independent on the molecular shape if monitored at multiple angles (as in MALLS). Because the CD data demonstrates that rLCC is not disordered (see below), the small elution volume indicates that the protein has an elongated shape, which is consistent with the structure of this fragment in the native laminin trimer.

Still the measured mass is larger than the theoretical mass of a non-glycosylated trimer, which is 253 kDa. Since laminin is heavily glycosylated and the majority of the potential glycosyilation sites are in the LCC domain [14], this mass excess is likely due to the sugar moiety of the molecule. This is consistent with the molar masses observed in the SDS-PAGE, which are larger than calculated for the individual chains (Fig. 2A). Therefore, the light scattering measurements and the Western blots are consistent with the presence of an α1β1γ1 heterotrimer.

The structure of the heterotrimer, investigated by circular dichroism, shows the spectrum has two minima at around 209 and 218 nm and a maximum at 192, very close to the spectral extrema typical of helical structures. The protein is globally folded into a stable three dimensional structure as seen by the cooperativity in the thermal denaturation followed by the decrease in the ellipticity at 222 nm, with a mid-point temperature of 58°C (Fig. 2B). These results indicate that the truncated laminin chains assemble into a stable heterotrimeric coiled-coil structure.

To further characterize the purified heterotrimer we performed additional ELISA experiments. Plastic coated with rLCC111 was efficiently recognized by a polyclonal anti-laminin antibody and by the scFv antibody L36, that recognizes a conformational epitope located in the middle part of the LCC [5]. As expected, control polyclonal and scFv antibodies did not recognize immobilized rLCC111 (Fig. 3). The presence of least two truncated laminin chains on the same rLCC111 molecule was further assessed by sandwich ELISA. Purified rLCC111 was added to wells pre-coated with anti-Flag (tα1 chain) or anti-HA (tγ1 chain) mAbs and, after washing, bound rLCC111 was detected by a conjugated anti-His (tβ1 chain) mAb (Fig. 3C).

**Figure 7:**
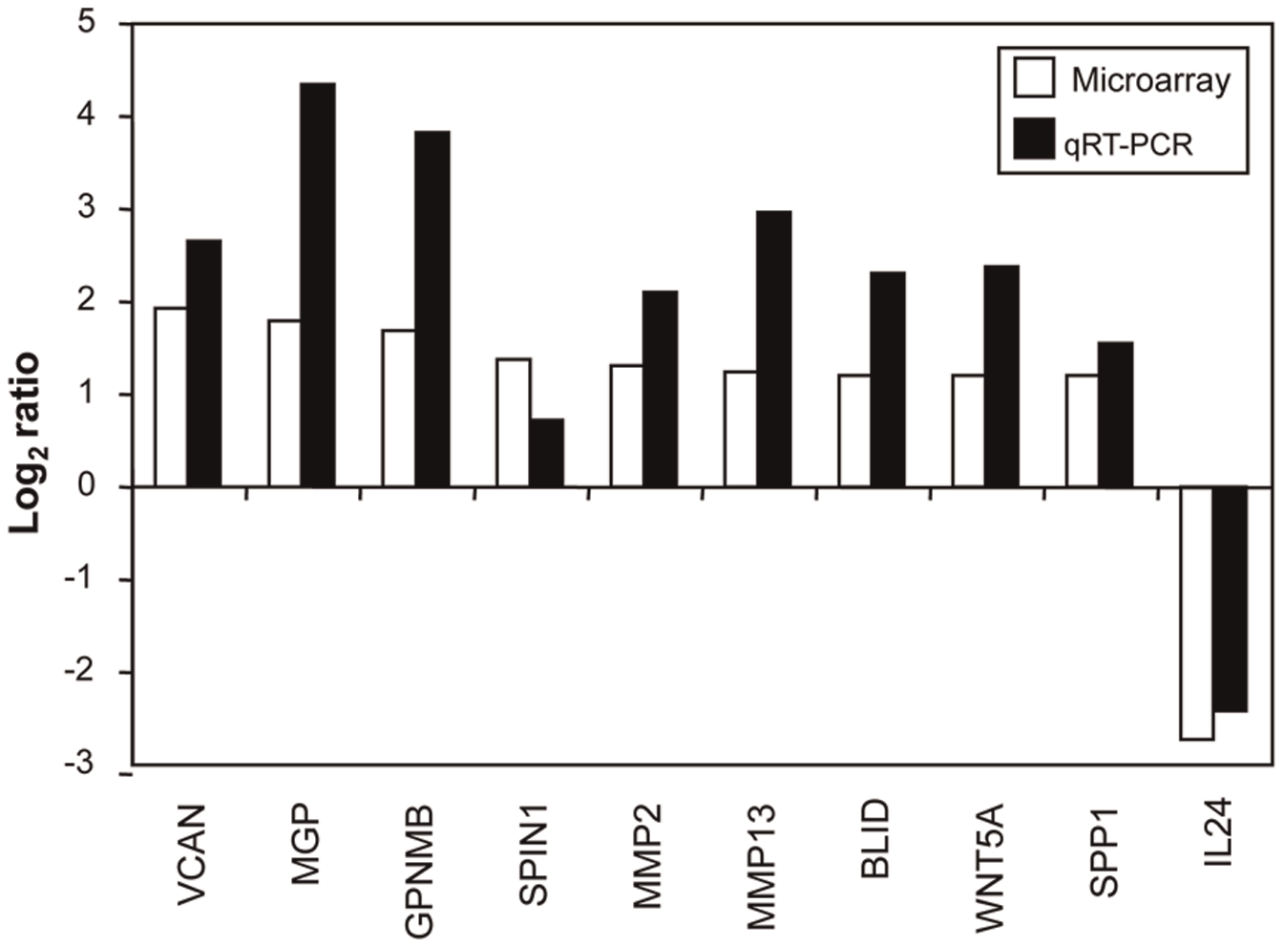
qRT-PCR analysis of genes selected from the microarray profile. Expression changes of 10 selected genes were evaluated in relation to the values obtained in parallel for SHDA. Log2 scale of fold change was used to show the relative expression value of each gene. Positive values indicate that the mRNA level of a particular gene was up-regulated, whereas negative values indicate that the transcript was down-regulated.

### Cell Adhesion and Spreading Activities of the rLCC111

Adhesion of HT1080 cells was examined by inoculating cells onto plastic plates precoated with different concentrations of native LM111 or rLCC111 (ranging from 0.08 to 20 μg/ml), or BSA. As expected, HT1080 cells adhered to intact LM111 in a concentration-dependent manner, but rLCC111 not only did not promote adhesion, but also showed a clear anti-adhesive effect (Fig. 4A), despite efficiently plastic coating (Fig. 4B). At higher coating concentrations (from 2.3 to 21 mg/ml) of both LM111 and rLCC111, statistically relevant differences were found (Fig. 4A). We next tested the effect of soluble rLCC111 on attachment of HT1080 cells to LM111 substrates. Addition of soluble rLCC111 at a concentration of 20 μg/ml at the time of cell plating resulted in a 50% reduction of cell attachment, while BSA at the same concentration had no effect (Fig. 4C).

The interaction of rLCC111 with membrane receptor molecules was further studied by immunofluorescence methods. As seen in Figure 5, both LM111 and rLCC111 bound to the HT1080 cell surface. Confocal microscopy revealed prominent cluster formation in cells incubated in the presence of rLCC111, whereas cells incubated with LM111 showed a more dispersed distribution (Fig. 5).

Actin cytoskeleton was visualized with fluorescently labeled phalloidin after plating HT1080 cells on BSA-, LM111- or rLCC111-substrates. Cells plated on LM111 substrate formed extensive stress fibers that traversed the cell body, that were absent in cells plated on BSA substrate. However, in cells seeded on rLCC111, actin staining was mainly localized to the cell periphery, including membrane ruffles and patch-like structures at the edge of the cells (Fig. 6A).

To characterize the effect of rLCC111 on cell morphology, cells were observed under a phase-contrast microscope at different periods of time after plating. Rapid and extensive spreading of HT1080 cells was induced on intact LM111, and most of the cells were spread after 1 h at 37°C. In contrast, cells retained a rounded morphology on rLCC111-coated plates and did not spread after 3 h (Fig. 6B and 6C).

### Microarray Analysis of Differentially Expressed Genes in HT0180 Cells Cultured on rLCC111-coated Wells

To study the effects of this interaction on the transcriptional profile of HT1080 cells, we performed a comprehensive DNA microarray analysis, using GeneChip® Affymetrix Human Gene 1.0 ST Arrays after 24 h-incubation in wells coated with BSA (control) or rLCC111. After restricting the profile to those sequences exhibiting ≥2-fold expression differences in rLCC111-treated relative to control samples, we identified 45 up-regulated and 17 down-regulated genes (Table S3). Of interest, several of the upregulated genes were components of the ECM with a role in the regulation of cell motility in physiological or pathological conditions. The most relevant are: MMP-2 (gelatinase A); MMP-13 (collagenase 3); VCAN (versican) and SSP1 (secreted phosphoprotein 1, osteopontin). DAVID clustering analysis confirmed that genes involved in ECM were significantly enriched and also revealed a high enrichment in genes implicated in ligand-mediated signaling and cell communication (Table S4).

### Validation of Microarray Data by qRT-PCR Analysis of Selected rLCC111-modulated Genes

To validate the expression microarray results by an independent gene expression profiling method, HT1080 mRNA levels for a subset of rLCC111-modulated genes were quantified by qRT-PCR (Fig. 7), using the primer pairs listed in Table S2. For this analysis, we selected 10 genes involved in matrix remodeling, cell motility and tumor progression. SHDA gene was used for data normalization. Primer specificity was assessed by sequencing. The direction of change in expression was in agreement by both qPCR and microarray for 100% of samples [18] considering a result valid if the fold change measured by both qPCR and microarray were greater than or equal to 2-fold. This criteria was fulfilled by all genes but OCR1. For the majority of the genes, fold-differences were also of comparable magnitude, although they were higher according to qRT-PCR for eight genes (VCAN, MGP, GPNMB, MMP2, MMP13, BLID, WNT5A and SSP1). However, for other genes, such as OCR1 and IL24, higher expression differences were detected by microarray. These results suggest that the interaction with rLCC111 induces in HT1080 cells a transcriptional profile compatible with a more invasive phenotype.

### Induction of MMP-2 Activation by rLCC111

**Figure 8:**
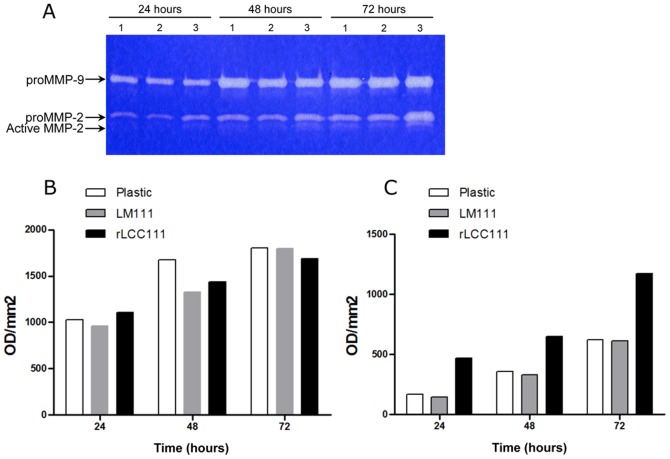
Representative gelatin zymogram showing MMP9 and MMP2 activities in HT1080 cells were cultured under serum free conditions for 72 hours under substrate-independent conditions on uncoated wells (1) or on LM111 (2) or rLCC111 (3) coated wells (A). Densitometric evaluation of MMP-9 (B) and latent MMP-2 (C).

To validate the functional significance of rLCC111-modulated genes, HT1080 cells were seeded on plastic, LM111, or rLCC111 and cultured for 3 days. The conditioned media was collected every 24 h and analyzed by zymography. HT1080 cells cultured on plastic constitutively synthesized and secreted latent MMP-2 and MMP-9. This pattern was similar to that observed in cells cultured on LM111 substrates (Fig. 8). In contrast, both active and latent MMP-2 were upregulated when HT1080 cells were cultured on rLCC111-coated wells (Fig. 8).

## Discussion

In this study we generated truncated laminin α1-, β1- and γ1-chains encoding the entire LCC. When expressed alone, the truncated α1 chain was found to be efficiently secreted as a monomer; while the truncated β1- or g1-chains were poorly secreted. However, if truncated α1-, β1- and γ1-chains were simultaneously expressed, then all three assembled into a trimer, which was secreted into the medium. The data confirm previous results showing that α-chain subunit expression is essential for secretion of β- and γ-chain partners [16].

Western blot analysis of the purified protein and the elution of a single peak in the size exclusion indicate that the three truncated α1-, β1- and γ1-chains assemble into a homogeneous multimeric structure. The MALLS measurements indicate that a heterotrimer is formed, and the circular dichroism spectrum displays the typical features of proteins cooperatively folded with predominantly helical structure. The discrepancy in the calculated and measured masses of the heterotrimer must be due to the glycosylation at multiple sites in the three chains. Although coiled-coil structures can be recognized in proteins by a minimum at 222 nm more negative than he one at 208 nm [19] this is only valid for proteins that are close to 100% coiled-coil, while in the rLCC111 there are several regions that are not: the chain ends, the Lβ domain in the β1 chain (also known as knot domain) and possibly other uncharacterized ones [20]. The formation of a coiled-coil structure can be indirectly inferred from the predominantly helical structure of the molecule and its elongated shape that causes it to elute from the size exclusion column at a volume corresponding to a three-fold larger apparent molecular mass.

Our results confirm that the truncated chains can assemble into the trimeric coiled-coil structure independently of the rest of the molecule. This is consistent with previous conformational analysis of the elastase produced laminin fragment E8 (containing approximately the C-terminal half of the coiled-coil domain plus three of the globular LG domains in the α1 chain) [21]. It is also consistent with the conformational studies of approximately 50-residue long peptides spanning the C-terminal ends of the coiled-coil domain [22], [23]. The circular dichroism spectrum of the equimolar mixture of these peptides showed a helical structure but with a less cooperative thermal denaturation than the rLCC111 fragment.

Laminins exert multiple biological functions through interactions with other ECM molecules and cell surface receptors [7]. However, most of the cell binding sites map to regions distinct from the coiled-coil domain. It is known that this domain plays a key role in chain assembly, but with few exceptions [5], [6] it has been considered a functionally silent domain. Here we demonstrated for the first time that the laminin coiled-coil domain inhibits cell adhesion and spreading.

Anti-adhesive properties of ECM proteins are not frequently observed [24]. Examples of anti-adhesive proteins include thrombospondin, SPARC (BM-40/osteonectin), and tenascin. The modulation of cell adhesion is often related to cell migration. Filopodia and lamellipodia formation is crucial for cell movement, and the actin cytoskeleton plays an important role in these processes [25]. As decreased actin stress fiber formation and increased membrane ruffle formation are consistent with the promotion of cell migration, it is tempting to speculate that the anti-adhesive property of the laminin coiled-coil domain is linked to the modulation of cell migration in tissues where the laminin coiled-coil domain became accessible.

In fact, whole genome expression profiling of HT1080 cells cultured on rLCC111-coated wells revealed the transcriptional regulation of a series of genes compatible with a pro-migratory and pro-invasive phenotype. MMP-2 is well known as a major contributor to the proteolytic degradation of ECM, playing a pivotal role in cell migration during physiological and pathological processes [26]. Recently, a role of MMP-13 in promotion of bone metastasis [27] and breast cancer invasiveness [28] has been reported.

Several ECM components whose expression is induced by rLCC111 are implicated in the regulation of cell motility. Osteopontin (SSP1) is a prominent matricellular protein whose aberrant expression has been associated with tumor development and progression [29], [30]. Even if it cannot be properly considered a matricellular protein, the proteoglycan versican (VCAN) is an ECM component able to regulate similar cellular processes [31]. Interestingly, IL-24/MDA-7 (Melanoma differentiation associated gene-7) expression is down-regulated in rLCC111-treated cells. IL-24 acts as tumor suppressor whose ectopic production has been reported to inhibit invasion and migration of human cancer cells [32], [33]. Other rLCC111-upregulated genes associated with increased metastasis are WNT5A [34], and OCR1 [35]. To our knowledge, full genome transcriptome analysis of cells cultured in the presence of laminin (intact molecule or fragments) has not been reported. Soluble protease-digested EHS-laminin (LM111) has been shown to drive transcriptional regulation in human breast cancer cells, as assessed by PCR arrays. The resultant gene expression pattern suggested the increase of cancer cell invasive capacity [36].

It is well established that proteases involved in tissue remodeling generate neoepitopes from ECM components that induce changes in cell behaviour. The most widely accepted interpretation is that those neoepitopes act as “soluble” ligands in the pericellular microenvironment that induce changes in cell migration and invasion [37]–[39]. We propose that in intact laminin molecules the coiled-coil-associated “anti-adhesive regions”, are cryptic or more likely their function is counteracted by “pro-adhesive regions”, located in the adjacent globular domains. The proteolytic processing of laminin and the release of N- and C-terminal globular fragments, would allow the functional predominance of coiled-coil-associated anti-adhesive regions. It has been shown that the specific cleavage of laminin-5 (LM322, α3β2γ2-subunit composition) by MMP-2 induces the migration of breast epithelial cells [40]. The cleavage site immediately precedes two closely spaced cysteines in domain LEB of the γ2 chain, which are involved in joining α3-, β2- and γ2-chains at the center of the cross. Importantly, whereas the N-terminal fragment of the MMP-2 cleavage site dissociated, the C-terminal fragment (γ2x) remains attached to the heterotrimer [40]. Therefore, the pro-migratory MMP-2 cleaved LM322 contains the entire LCC domain and the C-terminal LG domains of the α3 chain. This might explain the differences in cell adhesion, between the MMP-2 cleaved LM322 and the rLCC111 used in this study, lacking LG domains. There is evidence of EGF-like signaling initiated by the products of both MT1-MMP [41] and MMP-2 cleavage [42] of rat γ2 laminin short arm. In the study by Schenk et al [42], this activity is mapped to domain LEB, proposed as a cryptic migratory signal released by MMP action. These cleavage sites are not present in our truncated γ1 chain, that contains the C-terminal portion of domain LEB (two EGF-like modules). We cannot completely rule out the presence of alternative cleavage sites in t γ1, but we find it unlikely that potential cleaved fragments would contribute to rLCC111 activity.

The hypothesis that the LCC domain is less prone to proteolytic degradation than the rest of the molecule is supported by the recent identification of protease sensitive sites in LM111 using quantitative proteomics [43]. More than two thirds of the cleavage sites of four different proteases (including MMP-2, MMP-8 and MMP-9) were located outside the LCC domain. On this basis, we could envision a sequential cleavage of the laminin molecule, with the short arms being proteolyzed earlier whereas the LCC domain retains its integrity longer and exerts a defined functional effect.

In summary, we postulate that in specific pathological contexts, such as cancer progression, the degradome components in the tumor microenvironment could promote a local and temporal predominance of LCC moieties favoring a net invasive behaviour.

## Supporting Information

Figure S1 **Expression of individual truncated laminin chains in HEK-293 cells, transfected with empty plasmid (control) or plasmid encoding truncated mouse laminin α1 (tα1), β1 (tβ1) or γ1 (tγ1) chains.** Conditioned culture medium were collected and analyzed by Western blotting (A) and ELISA (B). Separated proteins on 8% polyacrylamide gels under reducing conditions were transferred onto nitrocellulose membranes followed by staining with anti-c-myc mAb. Simultaneous expression of truncated laminin chains in 293-F cells transfected with empty plasmid (control) or plasmids encoding truncated mouse laminin α1 (tα1), β1 (tβ1) or γ1 (tγ1) chains. Conditioned culture medium were collected and analyzed by Western blotting (C) and ELISA (D). Separated proteins on 4–12% gradient were transferred onto nitrocellulose membranes followed by staining with anti-Flag, anti-c-myc or anti-HA mAbs.
(TIF)

Figure S2 **SDS-PAGE and Western blotting analysis of the recombinant laminin coiled-coil domain (rLCC111).** Purified laminin from conditioned medium of 293-F cells, transfected with plasmids encoding tα1, tβ1 and tγ1 chains was analyzed on 4–12% gradient polyacrylamide gels under reducing conditions. Separated proteins were visualized by coomassie (A) or transferred onto nitrocellulose membranes followed by staining with anti-Flag, anti-c-myc or anti-HA mAbs (B).
(TIF)

Table S1 **Oligonucleotide sequence.**
(DOC)

Table S2 **Primer pairs used for real-time quantitative RT-PCR.**
(DOC)

Table S3 **Genes modulated (>2-fold) in HT1080 cells cultured on rLCC111** ***-*** **coated wells.**
(DOC)

Table S4 **DAVID enriched GO terms of differentially expressed genes between HT1080 cells cultured on BSA- or rLCC111** ***-*** **coated wells.**
(DOC)
