# Supplementary figures and images for: The Heterotrimeric Laminin Coiled-Coil Domain Exerts Anti-Adhesive Effects and Induces a Pro-Invasive Phenotype
Source: PLoS One. 2012 Jun 19;7(6):e39097. doi: 10.1371/journal.pone.0039097 (PMC3378518; doi:10.1371/journal.pone.0039097)

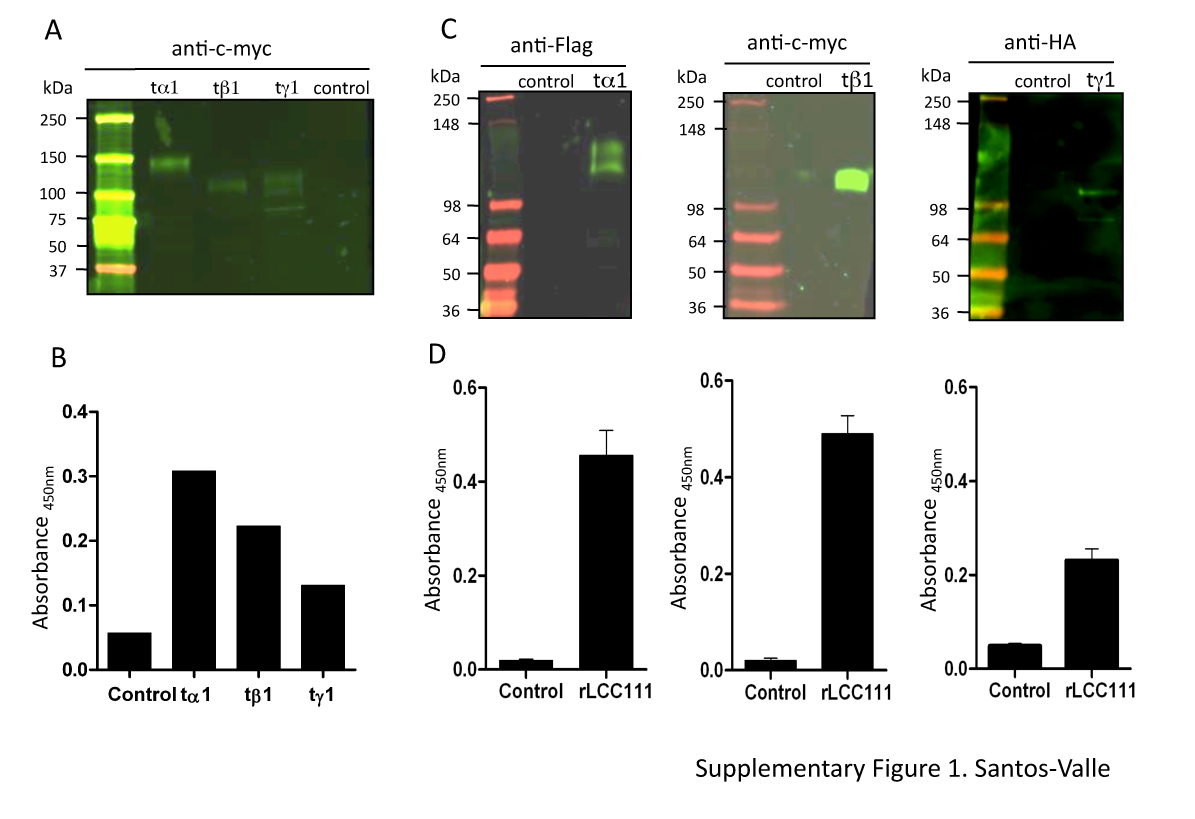

Supplement: Figure S1 — Expression of individual truncated laminin chains in HEK-293 cells, transfected with empty plasmid (control) or plasmid encoding truncated mouse laminin α1 (tα1), β1 (tβ1) or γ1 (tγ1) chains. Conditioned culture medium were collected and analyzed by Western blotting (A) and ELISA (B). Separated proteins on 8% polyacrylamide gels under reducing conditions were transferred onto nitrocellulose membranes followed by staining with anti-c-myc mAb. Simultaneous expression of truncated laminin chains in 293-F cells transfected with empty plasmid (control) or plasmids encoding truncated mouse laminin α1 (tα1), β1 (tβ1) or γ1 (tγ1) chains. Conditioned culture medium were collected and analyzed by Western blotting (C) and ELISA (D). Separated proteins on 4–12% gradient were transferred onto nitrocellulose membranes followed by staining with anti-Flag, anti-c-myc or anti-HA mAbs. (TIF) [file pone.0039097.s001.tif]

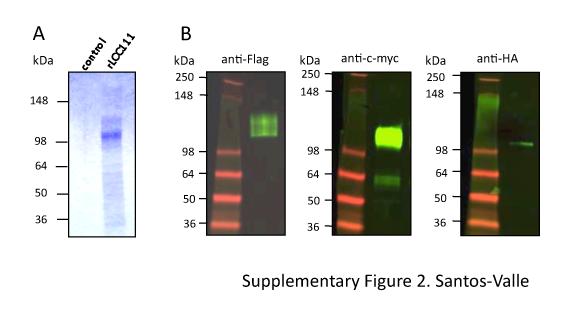

Supplement: Figure S2 — SDS-PAGE and Western blotting analysis of the recombinant laminin coiled-coil domain (rLCC111). Purified laminin from conditioned medium of 293-F cells, transfected with plasmids encoding tα1, tβ1 and tγ1 chains was analyzed on 4–12% gradient polyacrylamide gels under reducing conditions. Separated proteins were visualized by coomassie (A) or transferred onto nitrocellulose membranes followed by staining with anti-Flag, anti-c-myc or anti-HA mAbs (B). (TIF) [file pone.0039097.s002.tif]
