# Supplementary material for: The Heterotrimeric Laminin Coiled-Coil Domain Exerts Anti-Adhesive Effects and Induces a Pro-Invasive Phenotype
Source: PLoS One. 2012 Jun 19;7(6):e39097. doi: 10.1371/journal.pone.0039097 (PMC3378518; doi:10.1371/journal.pone.0039097)
Supplement: Table S1 — Oligonucleotide sequence. (DOC) [file pone.0039097.s003.doc]

**Table S1:** Oligonucleotide sequence^a^

| Name | Sequence (5´-3´) |
| --- | --- |
| **1a** | atcaaggtggcggccgcggtagatgaagtctgaggctgataggc |
| **2a** | ttgtaagctttgcaattgcaacaaccacagtgatgtctgt |
| **3b** | tactacagagaaaagctcccag |
| **4b** | aaacatcaaagaaatcacagaa |
| **5b** | aaggaaagtgtcggtaccagga |
| **6b** | ataaacaattcaacgctgatgt |
| **7c** | ggccgctgactacaaagacgatgacgacaagtgtaaatcgatt |
| **8c** | ctagaatcgatttatcacttgtcgtcatcgtctttgtactcagc |
| **9a** | ctgtggatcctgtgtctgcaattacctgggcacggtgaag |
| **10a** | atagtttagcggccgctaagcaggtgctgtaaaccgcaactttctc |
| **11b** | accacaacattgacactaccgatccaga |
| **12b** | aattaactgatacagcttcaca |
| **13b** | aaacttcctgactgaggatagt |

^a^Sequences of the primers applied for the construction of the truncated mouse laminin chains.

^b^Sequences of the primers applied for the verification of the laminin sequences.

^c^Sequences of the primers used for the insertion of the FLAG-tag sequence.
