# Supplementary material for: The Heterotrimeric Laminin Coiled-Coil Domain Exerts Anti-Adhesive Effects and Induces a Pro-Invasive Phenotype
Source: PLoS One. 2012 Jun 19;7(6):e39097. doi: 10.1371/journal.pone.0039097 (PMC3378518; doi:10.1371/journal.pone.0039097)
Supplement: Table S2 — Primer pairs used for real-time quantitative RT-PCR. (DOC) [file pone.0039097.s004.doc]

|  | Primer location  (5’ Pos) | Primer sequence (5´- 3´) | Product size |
| --- | --- | --- | --- |
| BLID | F 393  R 484 | GGCAGTTCCATTTATCCAGA  ACTGTTTCCTTAGGCAACAT | 92 |
| MMP13 | F 125  R 214 | TGAGGAAGACCTCCAGTT  CTTGCTGCATTCTCCTTCA | 90 |
| MMP2 | F 651  R 741 | GAGTTGGCAGTGCAATAC  GCATCTTCTTTAGTGTGTCC | 91 |
| IL24 | F 124  R 217 | CAAAGCCTGTGGACTTTAG  TAAAACCCAGGCAAGGGA | 94 |
| SPP1 | F 48  R 137 | CCCATCTCAGAAGCAGAAT  TCATCATCCATATCATCCATGT | 90 |
| VCAN | F 357  R 449 | GTGTCACTGACTGTGGAT  CAAACAAGCCTTCTGAGC | 93 |
| WNT5A | F 326  R 417 | GACATCGAAGGTGGAACT  GTATGTGAAGGCCGTCTC | 92 |
| GPNMB | F 123  R 215 | GGAGACATGAGGTGGAAA  TGTTATATTTGAGCCCACGAG | 93 |
| MGP | F 46  R 140 | ACAGGAGAAATGCAAATACC  CTCGTGGACAGGCTTAGA | 95 |
| SPIN1 | F 221  R 310 | GTTTCTGCGCTTGAAGTC  CACTGCTTTGCCAATCAT | 90 |
| SDHA | F 229  R 314 | TGGGAACAAGAGGGCATCTG  CCACCACTGCATCAAATTCATG | 86 |

**Table S2:** Primer pairs used for real-time quantitative RT-PCR
