# Supplementary material for: The Heterotrimeric Laminin Coiled-Coil Domain Exerts Anti-Adhesive Effects and Induces a Pro-Invasive Phenotype
Source: PLoS One. 2012 Jun 19;7(6):e39097. doi: 10.1371/journal.pone.0039097 (PMC3378518; doi:10.1371/journal.pone.0039097)
Supplement: Table S3 — Genes modulated (>2-fold) in HT1080 cells cultured on rLCC111 - coated wells. (DOC) [file pone.0039097.s005.doc]

**Table S3. Genes modulated (>2-fold) in HT1080 cells cultured on rLCC111-coated wells**

| **Gene name** | **Accession number** | **Fold change** |
| --- | --- | --- |
| Amelotin (AMTN) | NM_212557 | **10.162** |
| ST6 beta-galactosamide alpha-2,6-sialyltranferase 2 (ST6GAL2), | NM_032528 | **4.344** |
| ATPase, H+ transporting, lysosomal 38kDa, V0 subunit d2 (ATP6V0D2) | NM_152565 | **4.006** |
| Versican (VCAN) | NM_004385 | **3.863** |
| Matrix Gla protein (MGP) | NM_000900 | **3.551** |
| Tryptophan 2,3-dioxygenase (TDO2) | NM_005651 | **3.363** |
| Glycoprotein (transmembrane) nmb (GPNMB) | NM_001005340 | **3.261** |
| Plexin domain containing 2 (PLXDC2) | NM_032812 | **3.186** |
| Prostate transmembrane protein, androgen induced 1 (PMEPA1) | NM_020182 | **3.115** |
| Tumor necrosis factor, alpha-induced protein 6 (TNFAIP6) | NM_007115 | **3.089** |
| Taste receptor, type 2, member 4 (TAS2R4) | NM_016944 | **2.892** |
| Ovarian cancer-related protein 1 (OCR1) | AF314543 | **2.698** |
| Solute carrier family 2 (facilitated glucose transporter), member 12 (SLC2A12) | NM_145176 | **2.644** |
| Neural precursor cell expressed, developmentally down-regulated 9 (NEDD9) | NM_001142393 | **2.634** |
| G protein-coupled receptor 21 (GPR21) | NM_005294 | **2.593** |
| MicroRNA pri-miR-21, complete sequence | AY699265 | **2.549** |
| Platelet-derived growth factor receptor, beta polypeptide (PDGFRB) | NM_002609 | **2.540** |
| Matrix metallopeptidase 2 (gelatinase A) (MMP2) | NM_004530 | **2.518** |
| T-complex 11 (mouse)-like 2 (TCP11L2) | NM_152772 | **2.454** |
| Matrix metallopeptidase 13 (collagenase 3) (MMP13) | NM_002427 | **2.395** |
| BH3-like motif containing, cell death inducer (BLID) | NM_001001786 | **2.384** |
| Wingless-type MMTV integration site family, member 5A (WNT5A) | NM_003392 | **2.365** |
| Fibrillin 1 (FBN1) | NM_000138 | **2.349** |
| Metastasis associated lung adenocarcinoma transcript 1 (MALAT1) | BC018448 | **2.341** |
| EGF-like repeats and discoidin I-like domains 3 (EDIL3) | NM_005711 | **2.298** |
| Secreted phosphoprotein 1 (SPP1) | NM_001040058 | **2.284** |
| Mannosidase, beta A, lysosomal (MANBA) | NM_005908 | **2.277** |
| KIAA1199 | NM_018689 | **2.264** |
| Leucine rich repeat (in FLII) interacting protein 1 (LRRFIP1) | NM_001137550 | **2.245** |
| Taste receptor, type 2, member 3 (TAS2R3) | NM_016943 | **2.204** |
| Olfactory receptor, family 4, subfamily F, member 16 (OR4F16) | NM_001005277 | **2.203** |
| Transducer of ERBB2, 2 (TOB2) | BC028919 | **2.193** |
| SLIT and NTRK-like family, member 6 (SLITRK6) | NM_032229 | **2.165** |
| ATP-binding cassette, sub-family A (ABC1), member 1 (ABCA1) | NM_005502 | **2.163** |
| Potassium inwardly-rectifying channel, subfamily J, member 15 (KCNJ15) | NM_002243 | **2.161** |
| HtrA serine peptidase 1 (HTRA1) | NM_002775 | **2.134** |
| Solute carrier family 16, member 4 (monocarboxylic acid transporter 5) (SLC16A4) | NM_004696 | **2.083** |
| POTE ankyrin domain family, member C (POTEC) | NM_001137671 | **2.077** |
| POTE ankyrin domain family, member B (POTEB) | NM_207355 | **2.066** |
| Glypican 6 (GPC6) | NM_005708 | **2.065** |
| Unc-5 homolog B (Celegans) (UNC5B) | NM_170744 | **2.054** |
| ATP-binding cassette, sub-family C (CFTR/MRP), member 3 (ABCC3) | NM_003786 | **2.029** |
| hAWMS1 (TRA2A) | AB052759 | **2.025** |
| Solute carrier family 1 (glial high affinity glutamate transporter), member 3 (SLC1A3) | NM_004172 | **2.013** |
| Small nucleolar RNA, H/ACA box 75 (SNORA75), non-coding RNA | NR_002921 | **2.011** |
| Fibroblast growth factor 1 (acidic) (FGF1) | NM_000800 | **0.487** |
| Urothelial cancer associated 1 (UCA1), non-coding RNA | NR_015379 | **0.487** |
| Membrane protein, palmitoylated 4 (MAGUK p55 subfamily member 4) (MPP4) | NM_033066 | **0.486** |
| ChaC, cation transport regulator homolog 1 (Ecoli) (CHAC1) | NM_024111 | **0.468** |
| Lymphocyte antigen 6 complex, locus K (LY6K) | NM_017527 | **0.433** |
| Collagen and calcium binding EGF domains 1 (CCBE1) | NM_133459 | **0.432** |
| Transmembrane protein 71 (TMEM71) | NM_144649 | **0.424** |
| Lysophosphatidic acid receptor 1 (LPAR1) | NM_057159 | **0.420** |
| Gamma-aminobutyric acid (GABA) A receptor, alpha 6 (GABRA6) | NM_000811 | **0.411** |
| Small nucleolar RNA, C/D box 41 (SNORD41), non-coding RNA | NR_002751 | **0.406** |
| Aldehyde oxidase 1 (AOX1) | NM_001159 | **0.406** |
| Interleukin 8 (IL8) | NM_000584 | **0.386** |
| CD22 molecule (CD22) | NM_001771 | **0.381** |
| MAS-related GPR, member X4 (MRGPRX4) | NM_054032 | **0.359** |
| Serpin peptidase inhibitor, clade B, member 7 (SERPINB7) | NM_003784 | **0.323** |
| Endothelial cell-specific molecule 1 (ESM1) | NM_007036 | **0.268** |
| Interleukin 24 (IL24) | NM_006850 | **0.159** |
