# Supplementary material for: The Heterotrimeric Laminin Coiled-Coil Domain Exerts Anti-Adhesive Effects and Induces a Pro-Invasive Phenotype
Source: PLoS One. 2012 Jun 19;7(6):e39097. doi: 10.1371/journal.pone.0039097 (PMC3378518; doi:10.1371/journal.pone.0039097)
Supplement: Table S4 — DAVID enriched GO terms of differentially expressed genes between HT1080 cells cultured on BSA- or rLCC111 - coated wells. (DOC) [file pone.0039097.s006.doc]

**Table S4.** DAVID enriched GO terms of differentially expressed genes between HT1080 cells cultured on BSA- or rLCC111-coated wells

|  |  |  |  |  |  |  |  |  |
| --- | --- | --- | --- | --- | --- | --- | --- | --- |
| **Category** | **Term** | **Count/List** | **Pop Hits/Total** | **Fold Enrichment** | **PValue** | **Bonferroni** | **Benjamini** | **FDR** |
| **PANTHER_BP_ALL** |  |  |  |  |  |  |  |  |
|  | BP00122:Ligand-mediated signaling | 9/42 | 386/12713 | 7,0575 | 2,6658E-5 | 0,0020 | 0,0020 | 0,0279 |
|  | BP00274:Cell communication | 14/42 | 1125/12713 | 3,7668 | 3,1856E-5 | 0,0024 | 0,0012 | 0,0333 |
|  | BP00223:Angiogenesis | 3/42 | 52/12713 | 17,4629 | 0,0122 | 0,5954 | 0,2604 | 12,0129 |
|  | BP00102:Signal transduction | 18/42 | 3207/12713 | 1,6989 | 0,0165 | 0,7079 | 0,2648 | 15,9765 |
|  | BP00141:Transport | 9/42 | 1210/12713 | 2,2514 | 0,0369 | 0,9382 | 0,4269 | 32,5478 |
| **PANTHER_MF_ALL** |  |  |  |  |  |  |  |  |
|  | MF00178:Extracellular matrix | 11/42 | 352/13084 | 9,7351 | 9,3281E-8 | 5,7834E-6 | 5,7834E-6 | 9,4134E-5 |
|  | MF00181:Other extracellular matrix | 4/42 | 36/13084 | 34,6138 | 1,8978E-4 | 0,0117 | 0,0058 | 0,1914 |
|  | MF00179:Extracellular matrix structural protein | 4/42 | 82/13084 | 15,1963 | 0,0021 | 0,1239 | 0,0431 | 2,1290 |
|  | MF00016:Signaling molecule | 8/42 | 741/13084 | 3,3633 | 0,0076 | 0,3754 | 0,1110 | 7,3744 |
|  | MF00082:Transporter | 7/42 | 613/13084 | 3,5574 | 0,0115 | 0,5107 | 0,1332 | 10,9841 |
|  | MF00180:Extracellular matrix glycoprotein | 3/42 | 94/13084 | 9,9422 | 0,0349 | 0,8897 | 0,3075 | 30,1486 |
|  |  |  |  |  |  |  |  |  |
